# HIF-1α overexpression in mesenchymal stem cell-derived exosomes mediates cardioprotection in myocardial infarction by enhanced angiogenesis

**DOI:** 10.1186/s13287-020-01881-7

**Published:** 2020-08-28

**Authors:** Jiacheng Sun, Han Shen, Lianbo Shao, Xiaomei Teng, Yueqiu Chen, Xuan Liu, Ziying Yang, Zhenya Shen

**Affiliations:** Department of Cardiovascular Surgery of the First Affiliated Hospital & Institute for Cardiovascular Science, Soochow University, No.899, Pinghai Road, Suzhou, 215006 China

**Keywords:** HIF-1α, Exosome, Mesenchymal stem cell, Myocardial infarction, Angiogenesis

## Abstract

**Background:**

Myocardial infarction (MI) is a severe disease that often associated with dysfunction of angiogenesis. Cell-based therapies for MI using mesenchymal stem cell (MSC)-derived exosomes have been well studied due to their strong proangiogenic effect. Genetic modification is one of the most common methods to enhance exosome therapy. This study investigated the proangiogenic and cardioprotective effect of exosomes derived from hypoxia-inducible factor 1-alpha (HIF-1α)-modified MSCs.

**Methods:**

Lentivirus containing HIF-1α overexpressing vector was packaged and used to infect MSCs. Exosomes were isolated from MSC-conditioned medium by ultracentrifugation. Human umbilical vein endothelial cells (HUVECs) were treated under hypoxia condition for 48 h co-cultured with PBS, control exosomes, or HIF-1α-overexpressed exosomes, respectively. Then the preconditioned HUVECs were subjected to tube formation assay, Transwell assay, and EdU assay to evaluate the protective effect of exosomes. Meanwhile, mRNA and secretion levels of proangiogenic factors were measured by RT-qPCR and ELISA assays. In vivo assays were conducted using the rat myocardial infarction model. PBS, control exosomes, or HIF-1α-overexpressed exosomes were injected through tail vein after MI surgery. Heart function was assessed by echocardiography at days 3, 14, and 28. At day 7, mRNA and protein expression levels of proangiogenic factors in the peri-infarction area and circulation were evaluated, respectively. At day 28, hearts were collected and subjected to H&E staining, Masson’s trichrome staining, and immunofluorescent staining.

**Results:**

HIF-1α-overexpressed exosomes rescued the impaired angiogenic ability, migratory function, and proliferation of hypoxia-injured HUVECs. Simultaneously, HIF-1α-overexpressed exosomes preserved heart function by promoting neovessel formation and inhibiting fibrosis in the rat MI model. In addition, both in vitro and in vivo proangiogenic factors mRNA and protein expression levels were elevated after HIF-1α-overexpressed exosome application.

**Conclusion:**

HIF-1α-overexpressed exosomes could rescue the impaired angiogenic ability, migration, and proliferation of hypoxia-pretreated HUVECs in vitro and mediate cardioprotection by upregulating proangiogenic factors and enhancing neovessel formation.

## Background

Myocardial infarction (MI) is the leading cause for hospitalization and a major cause of death worldwide [1]. Restricted blood supply would cause irreversible loss of functional cardiomyocytes, which eventually leads to ventricular failure, resulting in significant alteration of life quality and mortality increment [2]. Therefore, many attempts have been made in recent years on promoting angiogenesis to stimulate the recovery of the microvasculature. Angiogenesis is the process of new blood vessel formation from pre-existing vasculature and is often dysregulated in MI [3]. The rescue of impaired angiogenesis is a prerequisite for therapeutic approaches.

Cell therapy using mesenchymal stem cells (MSCs) is a promising alternative therapeutic strategy [4]. MSCs can be rapidly mobilized from bone marrow and recruited to the ischemic heart, contributing to angiogenesis and cardiac repair [5]. Recent researches suggest that the effects of MSC are potentially mediated by extracellular vesicle (EV) [6], among which exosome, as a major subtype, has been substantially studied. Exosome is a nanosized particle that is released from the plasma membrane as a multivesicular body [7]. It has been considered as an important mediator of cell-cell communication, immunomodulation, proliferation, cell-senescence, and differentiation by transferring various bio-active cargoes such as mRNAs, microRNA, proteins, and lipids, from one cell to another [8–10]. Although exosome application exhibits a promising effect on angiogenesis in many animal models, such as myocardial infarction, wound healing, or limb ischemia [11–13], unmodified exosome only presents moderate therapeutic efficiency and needs to be enhanced by either genetic modification or engineering tools.

Hypoxia-inducible factor 1-alpha (HIF-1α) has been demonstrated as a key transcriptional regulator for gene expression in response to hypoxia [14–16]. Notably, HIF-1α regulates numerous gene expressions, including those encoding angiogenesis cytokines such as vascular endothelium growth factor (VEGF), platelet-derived growth factor (PDGF), and angiopoietin 1 (Ang-1) [17]. Li et al. transferred mutant HIF-1α, with delayed degradation, to ischemic rabbit skeletal muscle, and observed improved tissue perfusion, increased collateral vessels, and more histologically identifiable capillaries [18]. Gonzalez-King et al. also demonstrated that exosomes with overexpression of HIF-1α induced angiogenesis in the Matrigel plug assay via the enhanced expression of the Notch ligand Jagged1 [19]. However, the angiogenic effect of HIF-1α-overexpressed exosome remains unclear in ischemic heart.

In the present work, we investigated the effects of HIF-1α-overexpressed MSC-derived exosomes on hypoxia-injured HUVECs and ischemic heart. We showed that the migratory ability, angiogenic function, and proliferation of hypoxia-injured HUVECs were rescued by exosome application. Moreover, HIF-1α-overexpressed exosomes exhibited a strong cardioprotective effect on MI heart by promoting neovessel formation in the ischemic border zone.

## Methods

### Animals

Sprague Dawley rats, female, aged 3-week-old or 8-week-old, were housed at Animal Facility of Soochow University on a 12-h light/dark cycle with free access to water and standard rodent food. All animal procedures were approved by the Ethics Committee of Soochow University, Suzhou, China, and were carried out in accordance with the Guidelines for the Care and Use of Research Animals established by Soochow University.

### MSC culture and characterization

MSCs were derived from rats’ bone marrow and cultured in a standard incubator (Thermo Fisher Scientific, Marietta, OH, USA) as previously described [20]. Briefly, 3-week-old rats were sacrificed. Long bones, e.g., humerus, tibia, and femur, were collected by removing the attached muscle using a scalpel. Then, the bone marrow was thoroughly flushed from the bones using DMEM medium (Gibco, Grand Island, NY, USA) and passed through a 70-μm cell filter. The collected bone marrow was precipitated and washed by PBS twice. Finally, bone marrow mononuclear cells were seeded into cell dishes and cultured in DMEM medium supplemented with 10% fetal bovine serum (FBS; BI, Kibbutz Beit Haemek, Israel) and 1% of penicillin/streptomycin. The culture medium was changed every 2 days. Cells were digested with 0.25% trypsin (Gibco, Grand Island, NY, USA) and passaged when reaching 90% confluence. Passage 3 MSCs were used in all experiments.

MSCs were characterized by flow cytometry analysis [21]. When MSCs reach passage 3, they were collected and incubated with fluorescence-labeled antibodies against CD29, CD90, CD73, CD105, CD45, and CD11b. After incubation, cells were washed by 3% FBS/PBS before they were analyzed by a quantitative fluorescence-activated cell sorting (FACS) system (EMD Millipore, Burlington, MA, USA).

### Reconstruction of plasmids and lentivirus packing

The backbone vector pCDH-CMV-MCS-EF1-copGFP (SBI, Palo Alto, CA, USA) was used for the reconstruction of a lentiviral vector containing HIF-1α. HIF-1α fragment was amplified from rat total RNA using primers from Table 1. Note that EcoRI restriction endonuclease recognition site and Kozak consensus sequence were added on the forward primer, and BamHI restriction endonuclease recognition site was added to the reverse primer. Both PCR product and lentiviral vector were first digested with EcoRI and BamHI restriction enzymes (New England Bioscience, Ipswich, MA, USA) and then ligated by T4 ligase (Takara, Kusatsu, Japan). The reconstructed plasmid was verified by Sanger sequencing. For lentivirus packing, HEK293T cells (ATCC, USA) were co-transfected with a control vector or vector carrying HIF-1α fragment, and lentiviral packaging mix using Lipofectamine 2000 (Invitrogen, Carlsbad, CA, USA). Culture medium was collected 36 and 72 h after transfection, then passed through a 0.45-μm filter, and incubated with polyethylene glycol 8000 (PEG 8000) for 12 h at 4 °C before subjected to concentration at 4000×*g* for 20 min. Concentrated lentivirus was stored at − 80 °C. Genetic modified MSCs were used in the following assays 72 h after lentiviral infection. HIF-1α mRNA expression level in control MSCs (MSC^NC^) and HIF-1α-overexpressed MSCs (MSC^HIF-1α^) were measured by RT-qPCR.

**Table 1:** Primers sequences for HIF-1α amplification

Forward primer (5′–3′)	CGGAATTCGCCACCATGGAGGGCGCCGGCGGCGA
Reverse primer (5′–3′)	CGGGATCCTCAGTTAACTTGATCCAAAGCTCTG

### Cell viability assay

Cell viability after lentivirus transduction was evaluated by CCK-8 assay (Beyotime, Shanghai, China) as previously described [22]. Briefly, control MSCs (MSC group), MSCs transduced with control vector (MSC^NC^), and MSCs transduced with vector carrying HIF-1α fragment (MSC^HIF-1α^) were seeded in 96-well plates. The culture medium was changed to fresh medium with 10% CCK-8 solution 3 days after lentivirus transduction. The absorbance at 450 nm was measured in a Multi-Mode Microplate Reader (BIOTEK, USA).

### Exosome isolation

Exosomes isolated from the third passage MSC^NC^ or MSC^HIF-1α^ were referred as Exo or Exo-HIF-1α, respectively. When cells reached 90% confluence, the culture medium was discarded and cells were washed by PBS. Then, fresh DMEM medium, supplemented with 10% exosome-free FBS (exosomes were removed by ultracentrifuge at 120,000*×g* for 12 h [23]) and 1% penicillin/streptomycin, was added to culture dishes. After 48 h of culture, the conditioned medium was collected for exosome isolation. First, the collected medium was subjected to a centrifugation step of 400×*g* for 10 min at room temperature to remove cells. Next, supernatant was spun at 2000×*g* for 20 min at 4 °C to remove debris and apoptotic bodies. Then, the supernatant was centrifuged at 15,000×*g* for 40 min. Supernatant had passed through a 0.22-μm pore filter (Millipore, Burlington, MA, USA) before it was subjected to an ultracentrifugation step at 120,000×*g* for 70 min. Then exosomes were washed by a large volume of PBS before subjected to the second round of ultracentrifugation at 120,000×*g* for 70 min to purify exosomes. At no time during the process were samples subjected to temperatures below 4 °C. Exosome quantities were measured by bicinchoninic acid assay (BCA) using a BCA Protein Assay Kit (Beyotime, Shanghai, China) according to the manufacturer’s instruction. MSC-derived exosomes were identified by western blot, Nanoparticle Tracking Analysis (NTA), and transmission electron microscope (TEM). HIF-1α mRNA expression level in exosomes was measured by RT-qPCR.

### Quantitative RT-PCR assay

RT-qPCR samples include exosomes, MSCs, HUVECs, and the ischemic border zone of infarcted heart tissue. Total RNA was isolated from samples using TRIzol reagent (Invitrogen, Carlsbad, CA, USA), and reverse transcription was performed using the PrimeScript RT reagent kit (Takara, Kusatsu, Japan). The expression level of HIF-1α in exosomes, MSCs, HUVECs, and the ischemic border zone and the expression levels of VEGF, Ang-1, PDGF of HUVECs, and ischemic border zone were analyzed by SYBR Green assay following the manufacturer’s instruction, using GAPDH as control. Primers used are shown in Table 2. The 2^−ΔΔCT^ method was employed to determine the relative mRNA expression. Each assay was performed in triplicate.

**Table 2:** Primers sequences for RT-qPCR

Gene	Forward primer (5′–3′)	Reverse primer (5′–3′)
GAPDH	ATGACTCTACCCACGGCAAG	GGAAGATGGTGATGGGTTTC
Hif-1α	AGCAATTCTCCAAGCCCTCC	TTCATCAGTGGTGGCAGTTG
VEGF	GGGAGCAGAAAGCCCATGAA	AGATGTCCACCAGGGTCTCA
Ang-1	CACGACAGACCAGTACAACACAAACG	GACGACTGTTGTTGGTGGTAGCTCT
PDGF	CCGCTCCTTTGATGACCTTC	GCTCAGCCCCATCTTCGTC

### Western blot

Exosomes or MSCs were lysed with RIPA buffer containing protease inhibitor cocktail (Roche Applied Science, Penzberg, Germany). Extracted protein concentration was determined by BCA assay (Beyotime, Shanghai, China). Equal quantities of protein were loaded and run on 10% SDS-PAGE gels and then transferred to polyvinylidene difluoride (PVDF) membranes. Each membrane was blocked in 5% BSA and subsequently incubated overnight at 4 °C with anti-TSG101 (Abcam, UK) and anti-CD63 (Abcam, UK) antibodies for exosome characterization, anti-HIF-1α antibody (Abcam, UK) for MSCs analysis, and anti-Actin antibody (Beyotime, Shanghai, China) for both. After washing, the membranes were incubated with peroxidase-conjugated goat anti-rabbit or goat anti-mouse secondary antibodies (Invitrogen, Carlsbad, CA, USA). Image analysis and blot quantification were performed with Image Quant LAS 4000 mini biomolecular imager (GE Healthcare, Uppsala, Sweden).

### Nanoparticle tracking analysis

Exosome particle size and particle concentration were analyzed by nanoparticle tracking using a NanoSight LM10 system (NanoSight Ltd., Amesbury, UK). In brief, exosomes were diluted in particle-free PBS and introduced manually. Approximately 40–50 particles were in the field of view. Six videos were recorded for each independent measurement to generate data for each sample.

### Transmission electron microscope

Isolated exosomes were first resuspended in PBS and fixed with 3% glutaraldehyde solution for half an hour at room temperature. Then 20 ml sample was added to a copper grid and dying with 1% phosphotungstic acid for 5 min at room temperature. Imaging was performed on a Zeiss Libra 120 (Zeiss, Oberkochen, Germany) electron microscope at 120 kV.

### Exosome internalization

One micromolar of DiI lipophilic dye (Invitrogen, Carlsbad, CA, USA) was added to exosomes (250 μg) for exosome labeling. After 30 min of incubation at 37 °C, the excess dye was removed, and labeled exosomes were re-isolated by ultracentrifugation (described above). Recipient HUVECs (3 × 10^5^) were incubated with DiI-labeled exosomes (10 μg) for 2 h, then fixed in 4% paraformaldehyde (PFA) for 10 min at room temperature, washed with PBS for three times, and incubated with DAPI (1:500, Invitrogen, Carlsbad, CA, USA) for 5 min at room temperature. Samples were observed via fluorescence microscopy (Olympus, Japan).

### Hypoxia preconditioning of HUVECs in vitro

HUVECs were cultured in EGM-2 MV Single Quotes (Lonza, Basel, Switzerland) supplemented with 5% FBS. For hypoxic culture, HUVECs were cultured in a standard incubator composed of 94% N_2_, 5% CO_2_, and 1% O_2_ for 48 h. Four groups of HUVECs were used in the in vitro assays: HUVEC group (un-treated HUVECs), hypoxic HUVEC group (hypoxia-treated HUVCEs), hypoxic HUVECs + Exo group (hypoxia-treated HUVCEs co-cultured with exosomes derived from MSC^NC^), and hypoxic HUVECs + Exo-HIF-1α group (hypoxia-treated HUVCEs co-cultured with exosomes derived from MSC^HIF-1α^). The expression levels of HIF-1α, VEGF, Ang-1, and PDGF were measured using RT-qPCR. The secretion levels of VEGF, Ang-1, and PDGF in conditioned medium were measured by ELISA assay.

### Tube formation assay

The in vitro angiogenic ability of HUVECs was tested by tube formation assay using Matrigel. 2 × 10^4^/well HUVECs of each group were seeded on the top of 100 μl Matrigel in a 96-well plate and incubated at 37 °C for 6 h. Representative photos of the tube structure were taken by an inverted microscope (Olympus, Japan). Tube length and tube mash number were analyzed by ImageJ software.

### ELISA assay

The levels of proangiogenic factors including VEGF, Ang-1, and PDGF in conditioned medium of HUVECs and animal circulation were determined using ELISA kits according to the manufacturer’s instructions. VEGF and PDGF ELISA kits were purchased from Multi Sciences Biotech (Hangzhou, China); Ang-1 ELISA kit was purchased from Solarbio Life Sciences (Beijing, China).

### Transwell assay

The migratory function of HUVECs was determined by a modified Boyden chamber [24]. In the upper chamber, 1 × 10^4^ HUVECs from each group were suspended in 200 μl DMEM medium supplemented with 0.2% FBS. The lower chamber contained 500 μl of DMEM medium supplemented with 15% FBS. After 4 h incubation, cells on the upper side of the chamber were carefully wiped off, and the cells on the lower side were fixed with PFA and stained with DAPI. Photos were taken using an invert fluorescent microscope (Olympus, Japan). The numbers of migrated cells were counted using ImageJ.

### EdU staining assay

The proliferation ability of HUVECs was assessed by BeyoClick™ EdU Cell Proliferation Kit with Alexa Fluor 555 (Beyotime, Shanghai, China). Briefly, cells from each group were seeded in a 6-well plate with 10 μM EdU reagent and cultured at 37 °C for 2 h. Excessive reagents were washed away by PBS. Cells were then fixed with 500 μl of 4% PFA and incubated with a permeable solution. Five hundred microliters of the Click reaction solution was added into each well and incubated at room temperature for 30 min protected from light, followed by incubation with Hoechst 33342 staining solution for 15 min. Finally, the EdU-positive staining cells were observed using an inverted fluorescent microscope (Olympus, Japan) and counted by ImageJ software.

### Myocardial infarction model and assessment of heart functions

Acute myocardial infarction was induced in SD rats. Briefly, female rats aged 8 weeks old were anesthetized with 160 mg/kg bodyweight pentobarbital by intraperitoneal injection. After anesthesia, thoracotomy was performed and the left ventricle was exposed. The left anterior descending artery was ligated between the pulmonary artery outflow tract and the left atrium. After MI surgery, these rats were randomly divided into 3 groups and treated with tail vein intravenous injection of 500 μl PBS (PBS group), 2 × 10^10^ particles of exosomes derived from MSC^NC^ suspended in 500 μl PBS (Exo group), or 2 × 10^10^ particles of exosomes derived from MSC^HIF-1α^ suspended in 500 μl PBS (Exo-HIF-1α group), respectively. Rats were anesthetized for echocardiography detection on days 3, 14, and 28 post MI, using the Vevo 2100 system (VisualSonics Inc., Canada) with an 80-MHz probe.

### Heart harvesting

Seven days after MI, 3 rats from each group were euthanized to collect hearts and circulation blood for proangiogenic factor measurement. At day 28, rats were anesthetized and underwent a systematic injection of Griffonia (Bandeiraea) Simplicifolia lectin 1 (Vector, 1 mg per rat) by direct cardiac puncture. Rats were euthanized after 10 min, and hearts were harvested and prepared for paraffin tissue sectioning after fixation with 4% PFA. All embedded tissues were sectioned for 5 μm thick and used in the following assays.

### Histological examination

Hematoxylin and eosin (H&E) staining was performed for histological analysis of infarcted heart tissue using the H&E staining kit (Solarbio, Beijing, China) according to the manufacturer’s instruction. To elucidate the severity of myocardial fibrosis, Masson’s trichrome staining was performed. The stained sections were used to measure the average ratio of fibrosis area to the entire LV cross-sectional area (percent fibrosis area) and the average ratio of fibrosis length to entire internal LV circumference (percent fibrosis length).

### Immunofluorescence staining

The sections were first stained with antibody to Griffonia (Bandeiraea) Simplicifolia lectin 1 (Vector, 1:100) and then washed with PBS and stained with Alexa Fluor 546 rabbit anti-goat IgG (Invitrogen, 1:1000) at RT for 1 h. Nuclei were counterstained with DAPI, and sections were mounted in the aqueous mounting medium. Images were examined using a fluorescent microscope (OLYMPUS, Japan).

### Statistical analysis

All data are expressed as mean ± standard deviation (SD). Comparisons between two groups were assessed by the Student *t* test. One-way ANOVA was used to compare among 3 or 4 groups. *P* < 0.05 was considered as statistical significance. Statistical analysis was carried out using the statistical software GraphPad Prism 8 (GraphPad Software, San Diego, CA, USA).

## Results

### Characterization of MSCs and exosomes

The morphology and surface markers of rat bone marrow-derived MSCs were identified. Representative images of passage 1 and passage 3 cells (Supplemental Fig. 1A) showed typical spindle-shape and the cells were adherent to plastic dishes. Flow cytometry showed the cells were positive for CD29, CD90, CD73, and CD105, while negative for CD45 and CD11b (Supplemental Fig. 1B). Then MSCs at passage 3 were used for lentiviral infection. As shown in Supplemental Fig. 1C, more than 90% of MSCs was successfully transduced with lentivirus. CCK-8 assay confirmed that lentivirus transduction did not affect cell viability (Supplemental Fig. 1D). Western blot confirmed that HIF-1α protein level was increased in MSC^HIF-1α^ (Supplemental Fig. 1E). Meanwhile, RT-qPCR also indicated that HIF-1α mRNA level had increased about 19.75-fold in exosomes from MSC^HIF-1α^ compared with exosomes from MSC^NC^ (Supplemental Fig. 1F).

Exosomes were then isolated from MSCs using the ultracentrifugation method. Nanoparticle tracking analysis showed the diameter of exosomes ranged from 50 to 200 nm, with a peak of 145.7 nm (Supplemental Fig. 2A). TEM images showed homogeneous morphology and classical cup-shape exosomes (Supplemental Fig. 2B). The isolated exosomes were positive for surface markers CD63 and TSG101, which are especially enriched in the membrane of exosomes [23], and negative for actin (Supplemental Fig. 2C). The HIF-1α expression level was also elevated in the Exo-HIF-1α group compared with the Exo group (Supplemental Fig. 2D). We also performed western blot to detect HIF-1α protein level in these 2 groups (data not shown): HIF-1α protein was identified in neither group, indicating the genetic modification only changed HIF-1α mRNA expression in exosomes.

### In vitro angiogenesis of HUVECs was impaired by hypoxia but reversed by exosome application

**Fig. 1:**
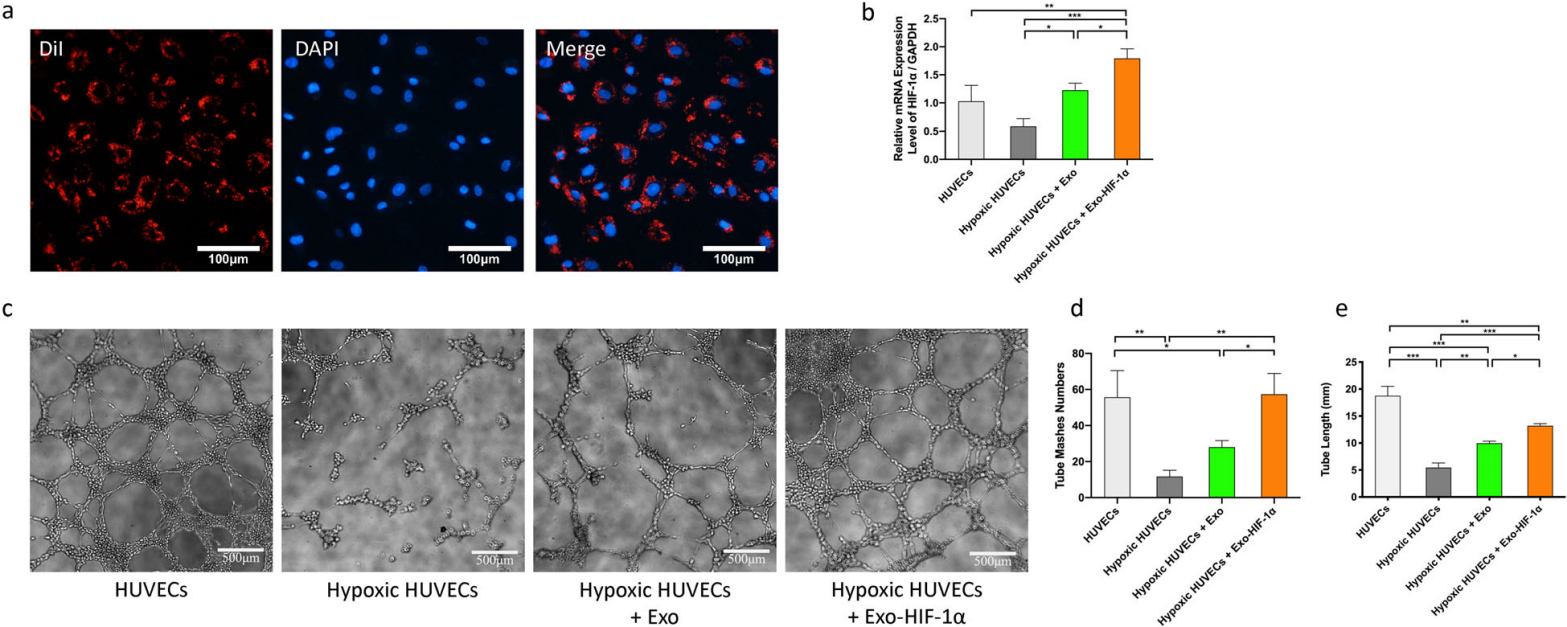
Exo-HIF-1α rescued the impaired angiogenesis of hypoxia-injured HUVECs. **a** Representative figures of HUVECs up-taking DiI-labeled exosomes. Bar: 100 μm. **b** HIF-1α mRNA expression level in 4 groups (*n* = 3). **c** Tube structure on Matrigel could be observed 6 h after seeding 4 groups of HUVECs. Bar: 500 μm. **d**, **f** Quantitative assessment of the total number of meshes number and tube length (*n* = 3). *P < 0.05, **P < 0.01, ***P < 0.001

To study the effects of exosomes on HUVECs, we first confirmed that HUVECs could take up exosomes by co-culturing CM-DiI dye-labeled exosomes with HUVECs (Fig. 1A). Then a hypoxia-injury model was established. After 48 h of co-culturing with PBS, Exo, or Exo-HIF-1α, HIF-1α mRNA level in 4 groups was measured. As shown in Fig. 1b, HIF-1α expression level was impaired after hypoxia precondition, but was rescued by Exo and Exo-HIF-1α application. Notably, Exo-HIF-1α increased HIF-1α mRNA level in hypoxia HUVECs by 1.74-fold compared with HUVECs in normoxic culture. This data indicated that exosomes could act as vehicles to trasfer HIF-1α mRNA into targeted cells.

We then tested the in vitro angiogenic ability of HUVECs. As shown in Fig. 1c, a large number of intact polygons formed by HUVECs was observed in the HUVECs group and hypoxic HUVECs + Exo-HIF-1α group. In contrast, hypoxic HUVCEs could barely form a polygonal structure, and hypoxic HUVCEs treated with Exo formed only few, incomplete tube-like structures. Quantitative figures of tube length and tube mash numbers were analyzed using ImageJ software and presented in Fig. 1d, e. Both tube length and tube mash numbers were significantly decreased after hypoxia culture (*P* < 0.001 for tube length; *P* < 0.01 for tube mashes). Although Exo administration elevated tube length by about 1.83-fold compared with PBS treatment (*P* < 0.01), this group failed to increase the tube mash number (*P* = 0.2467). Exo-HIF-1α co-culture elevated both tube length and tube mash numbers and presented a better protective effect than the Exo group.

### Expression levels of proangiogenic factors were upregulated by exosomes

**Fig. 2:**
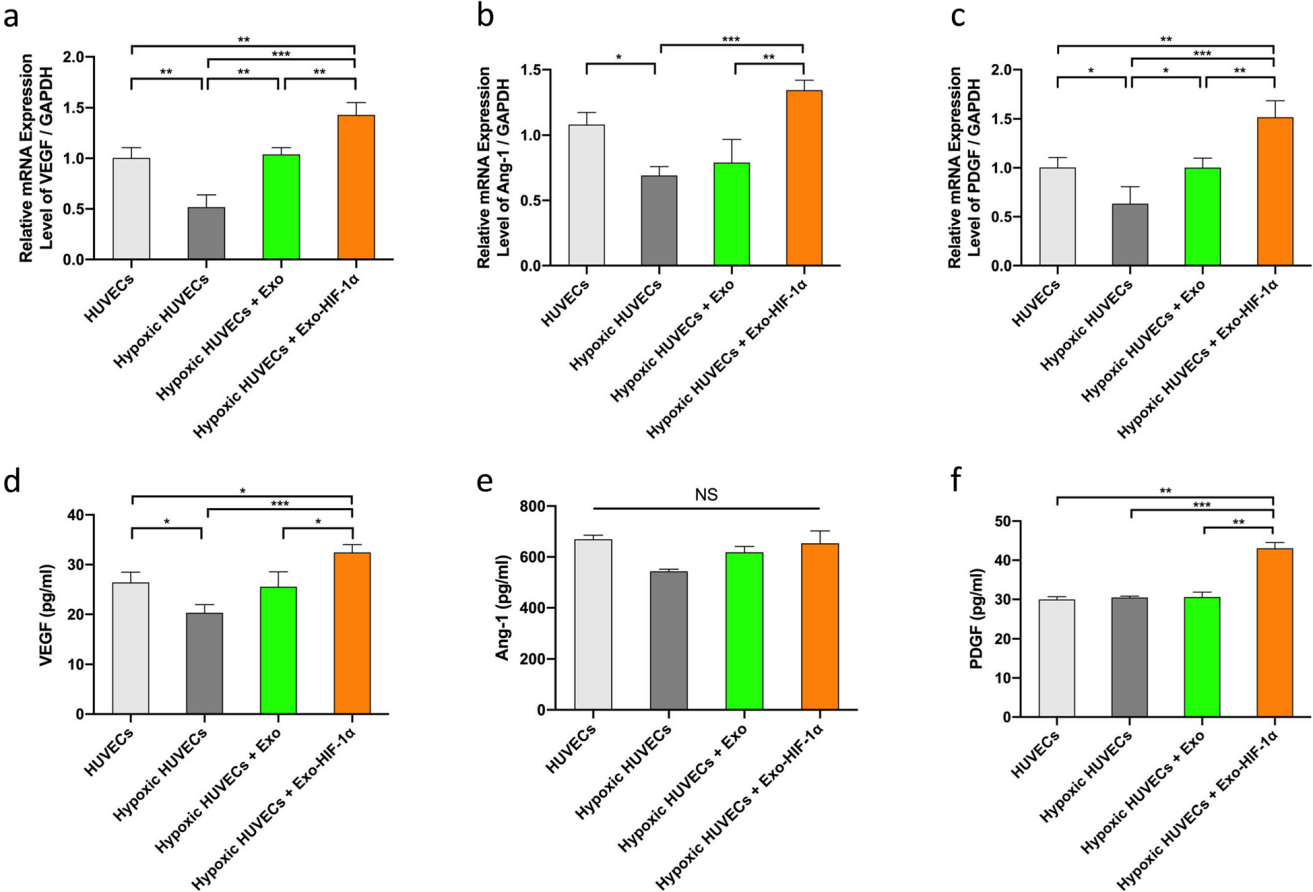
Exo-HIF-1α upregulated the downstream proangiogenic factors in hypoxia-injured HUVECs. **a**-**c** mRNA expression levels of VEGF, Ang-1, and PDGF in HUVECs, respectively (*n* = 3). **d**–**f** Protein expression level of VEGF, Ang-1, and PDGF detected by ELISA in the conditioned medium of HUVECs, respectively (*n* = 3). *P < 0.05, **P < 0.01, ***P < 0.001. NS, no significance

Further investigation of the genes encoding angiogenic factors provided a possible explanation for the aforementioned phenotype (Fig. 1). The mRNA expression levels of VEGF, Ang-1, and PDGF were all decreased by hypoxia injury. Exo-HIF-1α administration rescued the downregulated gene expression (Fig. 2a–c). However, ELISA assay suggested a partially consistent result. VGEF secretion presented a similar pattern as its mRNA level (Fig. 2d). Although Ang-1 secretion level showed a similar pattern as its mRNA level as well, there is no statistical significance among 4 groups (Fig. 2e). As in PDGF secretion level, hypoxia injury seemed to have no effect on PDGF mRNA translation. But HIF-1α could increase PDGF secretion in HUVECs (Fig. 2f).

### Migratory ability of hypoxia-preconditioned HUVECs was rescued by Exo-HIF-1α

**Fig. 3:**
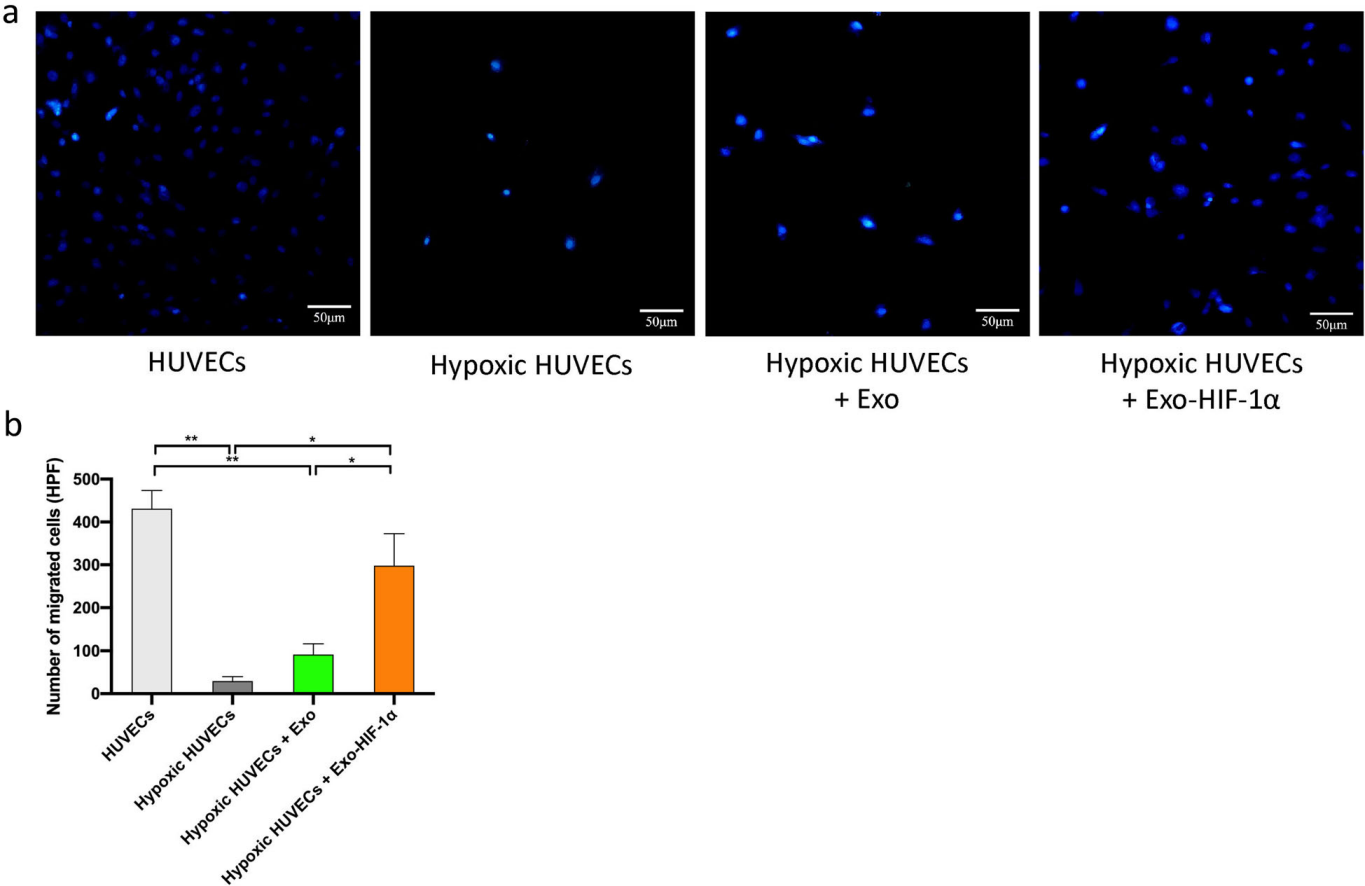
Migration of hypoxia-treated HUVECs was reversed by Exo-HIF-1α. **a** Representative figures of migrated HUVECs using a modified Boyden chamber. Bar: 50 μm. **b** Quantitative assessment of migrated cells per high power field (*n* = 3). *P < 0.05, **P < 0.01

Transwell system was introduced to study how exosomes regulate the migratory ability of HUVECs. As shown in Fig. 3, hypoxic culture for 48 h dramatically impaired the migratory ability of HUVCEs (HUVECs 431.00 ± 73.90/HPF vs. hypoxic HUVECs 29.49 ± 17.59/HPF; *P* < 0.01). However, co-culturing of either Exo or Exo-HIF-1α under hypoxic conditions could preserve the migratory ability. The Exo-HIF-1α group exerted greater protective effect than the Exo group (hypoxic HUVECs + Exo-HIF-1α 298.30 ± 129.2 vs. hypoxic HUVECs + Exo 91.27 ± 43.27; *P* < 0.05). In addition, although migrated cells in the Exo-HIF-1α group were less than the HUVEC group, statistical analysis presented no significance, indicating Exo-HIF-1α preserved the migratory ability of HUVEC during hypoxia injury.

### Proliferation of hypoxia-preconditioned HUVECs was augmented by Exo and Exo-HIF-1α

**Fig. 4:**
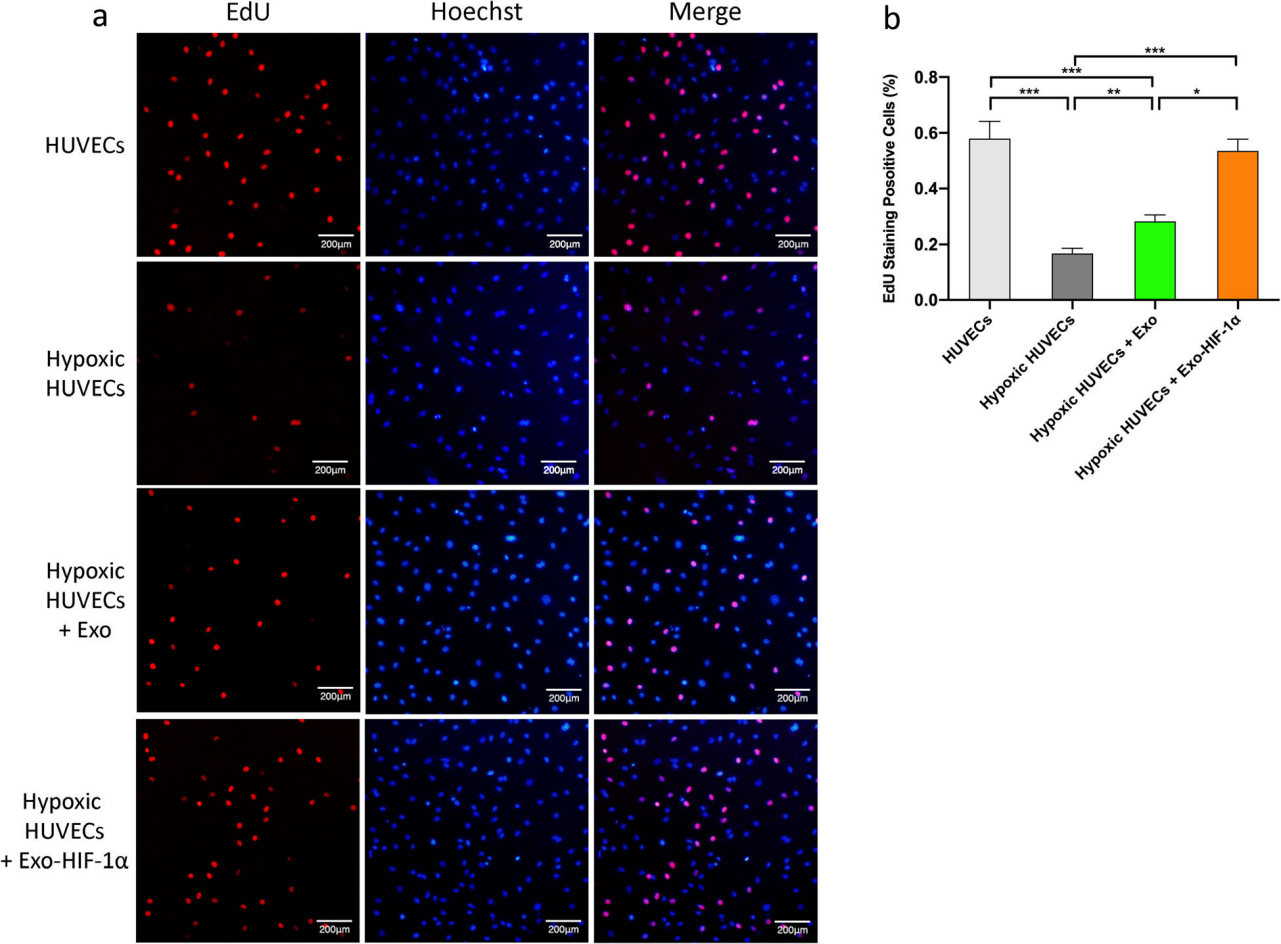
Proliferation of hypoxia-injured HUVECs was augmented by Exo-HIF-1α. **a** EdU has been incorporated into dividing HUVECs and been detected with a fluorescent azide using click chemistry. Bar: 200 μm. **b** The percentage of EdU positive cells was measured by ImageJ. *P < 0.05, **P < 0.01, ***P < 0.001

We performed an EdU staining assay to investigate the effects of different exosomes on preconditioned HUVECs proliferation (Fig. 4a). The ratio of EdU-positive cells dropped significantly after hypoxia preconditioning, while it was augmented after Exo or Exo-HIF-1α administration. Additionally, Exo-HIF-1α-treated hypoxic HUVECs presented similar EdU-positive ratio than the HUVEC group, indicating Exo-HIF-1α could completely preserve proliferation ability under hypoxia-induced injury (Fig. 4b).

### Exo-HIF-1α effectively preserved cardiac function in rat MI model

**Fig. 5:**
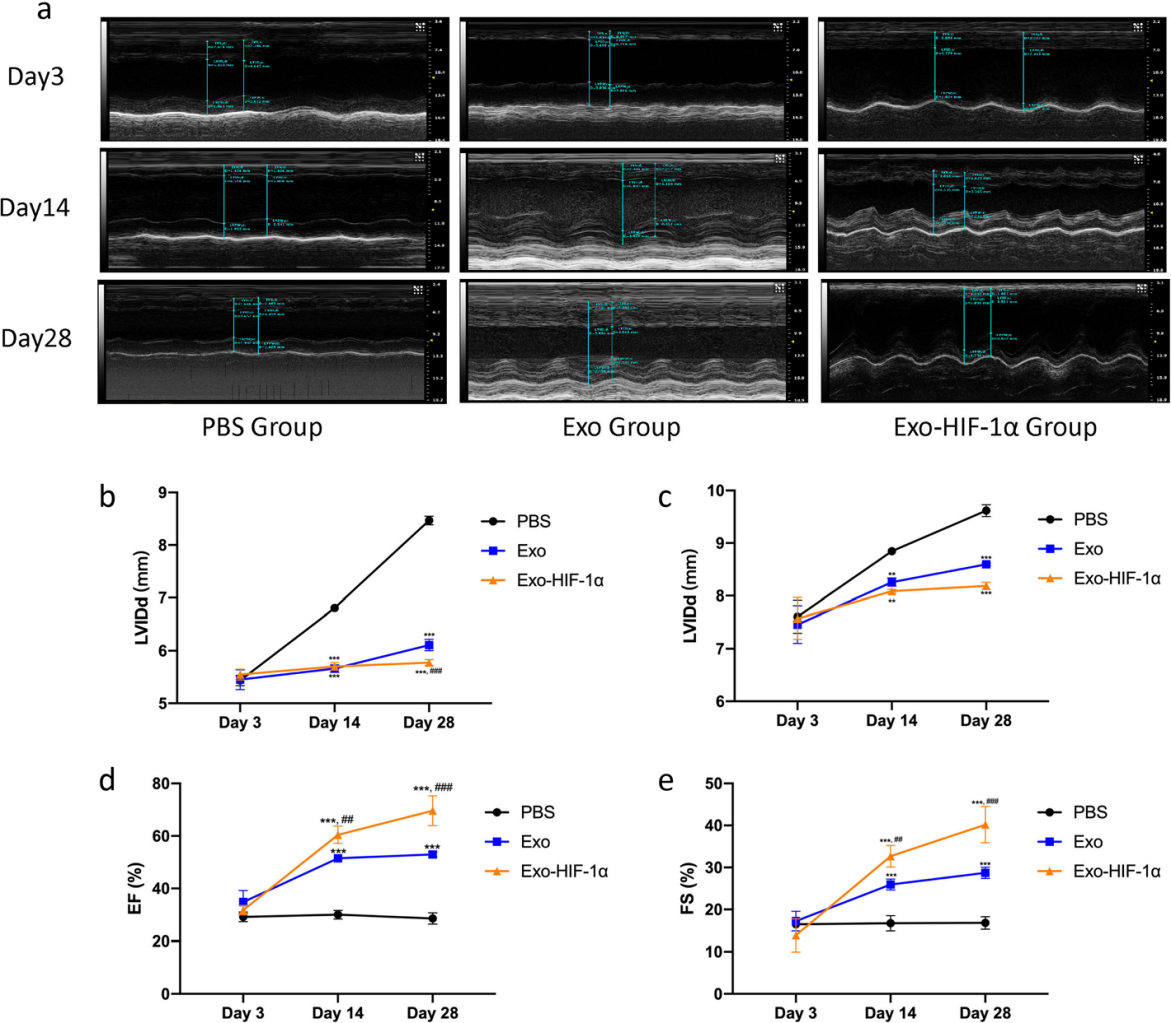
HIF-1α-overexpressed exosomes preserve cardiac function in the MI model. **a** Representative images of echocardiography. **b**, **c** left ventricular internal diameter end diastole and end systole (LVIDd and LVIDs) measured at day 3, day 7, and day 28 (*n* = 3). **d**, **e** left ventricular ejection fraction (EF) and left ventricular fractional shortening parameters (FS) in each group after MI at day 3, day 7, and day 28 (*n* = 3). **P < 0.01 vs. PBS group; ***P < 0.001 vs. PBS group; ^##^*P* < 0.01 vs. Exo group; ^###^*P* < 0.001 vs. Exo group

**Fig. 6:**
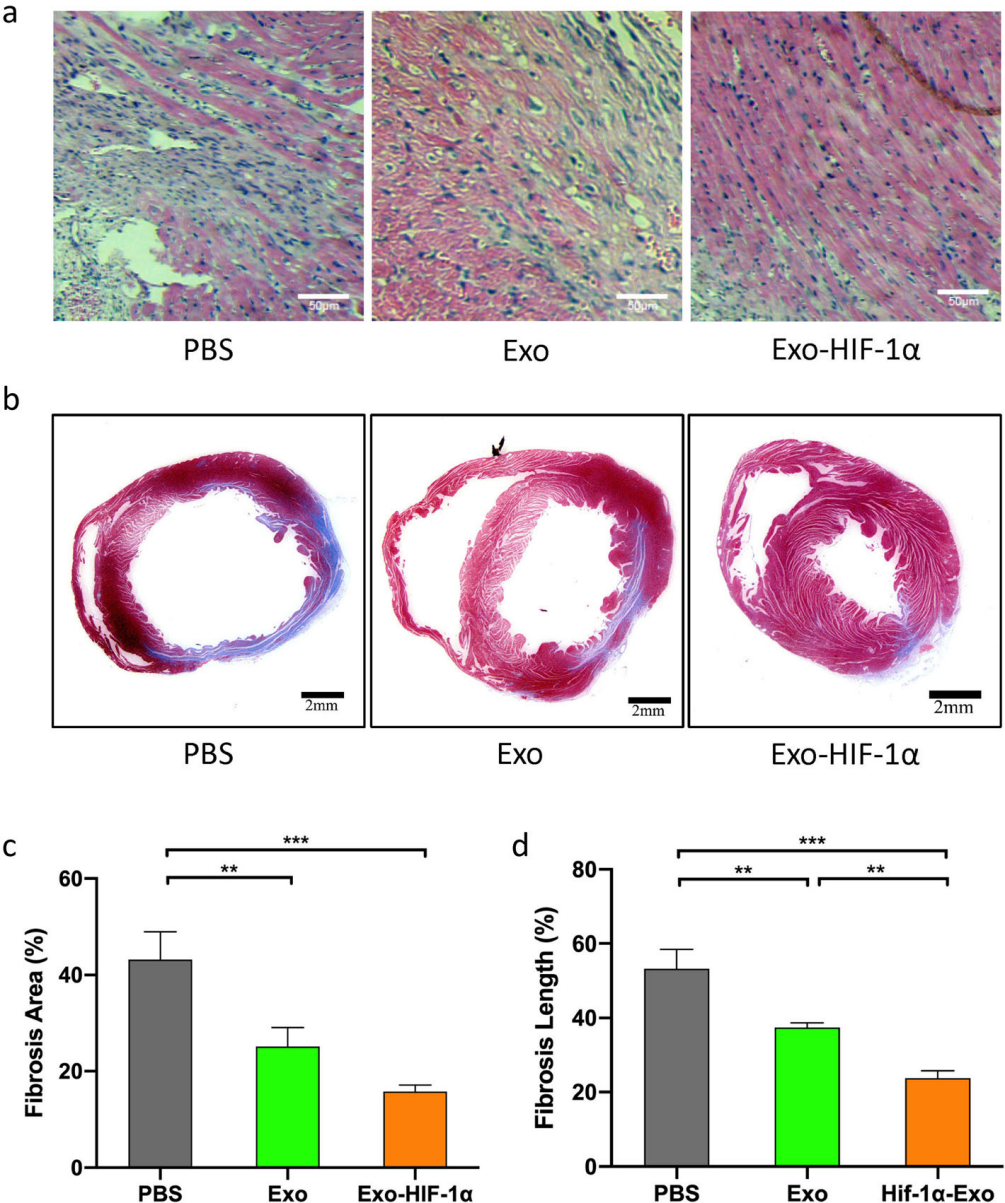
Histological assessment of the effect of exosomes on infarcted heart. **a** H&E staining showed different levels of fibrocyte infiltration in the ischemic area. **b** Masson trichrome staining of heart slides at 28 days after MI: red, myocardium; blue, scarred fibrosis. **c**, **d** Percentage of fibrotic area and length calculated and averaged by ImageJ software (*n* = 5). **P < 0.01, ***P < 0.001

Echocardiography was employed at 3, 14, and 28 days after cell transplantation to measure left ventricular internal diameter end diastole and end systole (LVIDd and LVIDs), left ventricular (LV) ejection fraction (EF), and LV fractional shortening parameters (FS) (Fig. 5a). LVIDd and LVIDs saw great increase in the PBS group in a time-dependent manner. However, Exo and Exo-HIF-1α application has significantly limited the expansion of the left ventricle and reduced cardiac remodeling (Fig. 5b, c). LVEF and LVFS were also increased in the Exo-HIF-1α group than the PBS group and the Exo group (Fig. 5d, e). This finding is consistent with our histological analysis (Fig. 6).

### Exo-HIF-1α reduced fibrosis in the infarcted heart

Next, we histologically assessed the protective effects of exosomes on myocardial infarction. From H&E staining, we observed that 28 days after MI, the infarcted heart underwent fibrosis and remolding to replace necrotic myocardial cells (Fig. 6a). Masson’s trichrome staining was also introduced to measure the size of infarcted tissue after 28 days. Representative images indicated that the fibrosis area was stained in blue and intact myocardium was stained in red (Fig. 6b). Quantitative analysis revealed that both the percent of fibrosis area in the LV cross-sectional area and the fibrosis length in internal LV circumference were significantly reduced in both the Exo and Exo-HIF-1α group (Fig. 6c, d). Exo-HIF-1α treatment presented better function in limiting the fibrosis length in the heart section.

### Angiogenesis was promoted in the infarcted heart by Exo-HIF-1α

**Fig. 7:**
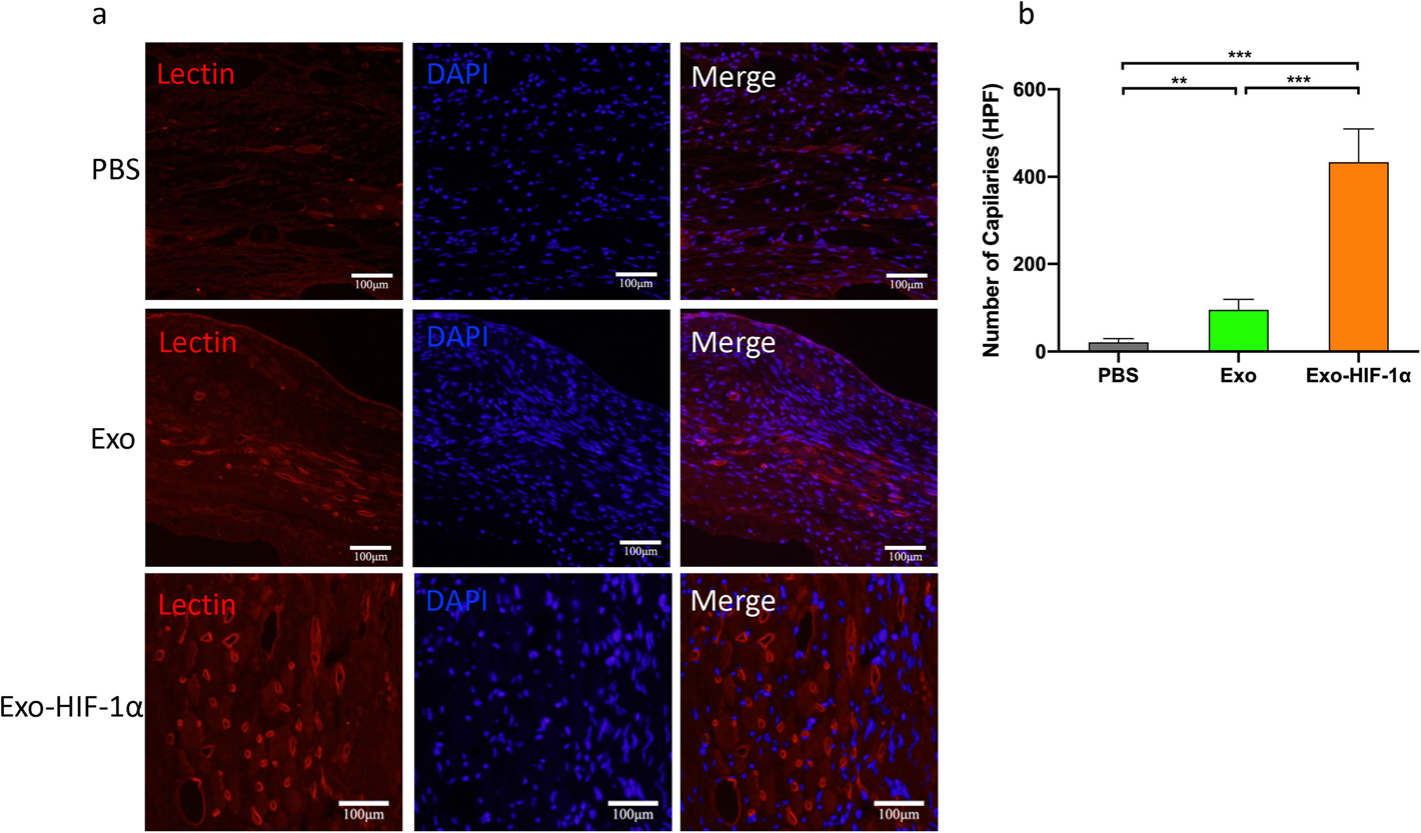
Neovessel formation in the infarcted border zone was detected by immunofluorescence staining. **a** Capillaries were visualized as tubular structure perfused by lectin. Bar: 100 μm. **b** Numbers of capillaries in each high-power field were manually counted (*n* = 3). **P < 0.01, ***P < 0.001

Angiogenesis in ischemic border zone was examined by immunohistochemistry. The immunofluorescent agent lectin was applied to mark the endothelial cells (Fig. 7a). Overall, the Exo-HIF-1α group exhibited enhanced angiogenesis ability compared with the PBS and Exo groups (Fig. 7b), indicating the cardiac protection induced by HIF-1α was mediated by its proangiogenic ability.

**Fig. 8:**
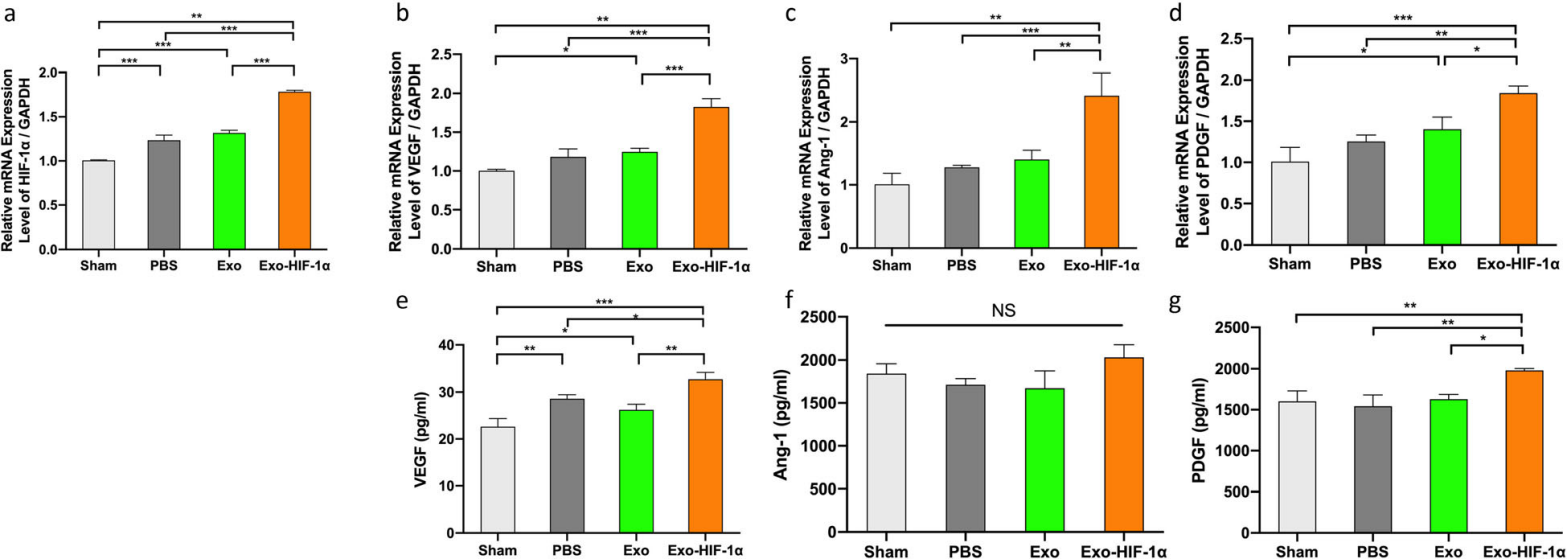
Different mRNA and protein expression levels in the infarcted border zone 7 days after myocardial infarction. **a**–**d** mRNA levels of HIF-1α, VEGF, Ang-1, PDGF, and SDF-1α were detected by RT-qPCR, respectively (*n* = 3). **e**–**g** Secretion levels of VEGF, Ang-1, and PDGF in circulation measured by ELISA, respectively (*n* = 3). *P < 0.05, **P < 0.01, ***P < 0.001. NS, no significance

The HIF-1α expression level in the PBS group and the Exo group has been upregulated mildly than the sham group, with 1.23-fold and 1.31-fold changes, respectively. While in the Exo-HIF-1α group, the HIF-1α expression level showed a significant increase compared with the other 3 groups (Fig. 8a). Further investigation revealed that most of the angiogenic mRNAs and proteins were elevated at day 7 post MI, showing consistency with the aforementioned immunohistochemistry finding. Three genes mentioned in our in vitro assay were all upregulated by HIF-1α application in the ischemic border zone (Fig. 8b–d). However, only VEGF and PDGF protein levels were increased by HIF-1α in rat circulation 7 days post MI (Fig. 8e, g). Ang-1 in the peripheral blood presented no statistical significance among 4 groups (Fig. 8f).

## Discussion

The present study demonstrated the cardioprotective effect of HIF-1α-overexpressed exosomes on rat myocardial infarction via enhanced angiogenesis and reduced fibrosis. Assays and data suggested that elevated HIF-1α expression level, induced by exosomes administration, led to the upregulation of proangiogenic growth factors both in vitro and in vivo. HIF-1α-overexpressed exosomes also exhibited promotional effects on migration and proliferation on hypoxia-injured HUVECs.

Dysregulation of angiogenesis and endothelial dysfunction are strongly associated with myocardial infarction [3, 25]. Inadequate angiogenesis usually results in a shortage of oxygen and nutritional supply and consequently leads to elevated cell death [26]. In light of the significant angiogenic potential, stem cell-based therapy in tissue regeneration and healing has been extensively studied. Among numerous cell-based tools, exosome shows great promise due to its high biocompatibility and low immunogenicity and, most importantly, its ability to induce revascularization, increase oxygen supply, inhibit cardiomyocyte apoptosis, and reduce myocardial fibrosis [11]. Our team has reported the therapeutic effect of MSC-derived exosomes on the microenvironment of infarcted myocardium via stimulated neovascularization and restrained inflammation response [27]. In addition, exosomes act as vehicles for horizontal transfer of bioactive molecules, such as mRNA, microRNA, and protein, in cell-to-cell communication [28]. Several studies suggested that, by gene overexpression strategy, exosomes derived from MSCs modified with protein kinase B (Akt) mRNA or C-X-C chemokine receptor type 4 (CXCR-4) mRNA could regulate the angiogenesis in myocardial infarction and exerted better therapeutic effects compared with exosomes derived from unmodified MSCs [29, 30]. In concordance with these previous studies, our research found that administration of exosomes or HIF-1α-overexpressed exosomes both rescued the impaired tube forming ability, migration ability, and cell proliferation of hypoxia injured HUVECs. In vivo assays presented similar effects: increased neovessel formation and decreased fibrosis was observed in the infarcted area, indicating the proangiogenic and cardioprotective effects of exosomes and HIF-1α-overexpressed exosomes. Notably, HIF-1α-overexpressed exosomes exhibited overall better therapeutic effects than control exosomes.

Hypoxia-inducible factor 1 (HIF-1) has been increasingly recognized for its key role in transcriptional control of more than a hundred genes which regulate a wide spectrum of cellular functional events, including angiogenesis, vasomotor control, glucose and energy metabolism, erythropoiesis, cell proliferation, and viability [31]. Since the first demonstration of the cardioprotective effect of HIF-1α by Cai et al. [32], accumulating evidence has suggested enhanced angiogenesis by HIF-1α plays a critical role in mediating cardioprotection [31]. Other studies suggested that HIF-1α could regulate the expression of many genes involved in angiogenesis, thus producing various physiological responses through different mechanisms to achieve mature functional neovascularization [33–35]. This current study showed uniformity with these previous reports: angiogenesis was enhanced by HIF-1α both in hypoxia-injured HUVECs and infarcted heart. Further investigation suggested the proangiogenic phenotype of HIF-1α was mediated by VEGF and PDGF. Ang-1 was ruled out due to no statistical significance in secretion level albeit an observed increasing trend in the Exo-HIF-1α group. The regulatory effect of HIF-1α on VEGF at the transcription level has been long elucidated [36]. HIF-1 upregulates the production of VEGF by binding to the hypoxia response element (HRE) in the VEGF promoter region. Several studies have also reported HIF-α induced increased capillary density as well as VEGF expression in peri-infarct and infarct regions [19, 37, 38]. Although direct involvement of HIF-1a in the regulation of PDGF gene expression is less reported, the proangiogenic effect of PDGF on endothelial cells is well documented. An earlier study showed that HIF-1a and PDGF cooperate with Akt signaling to mediate autocrine regulation which promotes angiogenesis in endothelial cells [39]. Another study reported an intriguing mechanism of an autocrine loop involving reactive oxygen species (ROS)/HIF-1a/PDGF in lung alveolar epithelial cells [40]. Taken together, our findings underpin the proangiogenic and cardioprotective role of exosome-delivered HIF-1a in infarcted heart via VEGF and PDGF.

## Conclusion

This study revealed the proangiogenic and cardioprotective effects of Exo-HIF-1α on ischemic heart were mediated via VEGF and PDGF. In addition, angiogenesis, proliferation, and migration of hypoxia-injured HUVECs were rescued by Exo-HIF-1α. In conclusion, HIF-1α overexpression in exosomes manifested therapeutic effects on myocardial infarction and could be envisioned as a promising therapeutic method for myocardial infarction.

## Supplementary information

**Additional file 1: Supplemental Fig. 1.** Characterization of mesenchymal stem cells (MSCs). (A): Morphology of MSCs (P1, P3) observed under microscope. Bar: 200 μm. (B): Cell surface antigens of MSCs, CD29, CD90, CD73, CD105, CD45 and CD11b, analyzed by flow cytometry. (C): Representative images of MSCs transduced with lentivirus containing HIF-1α were taken under an invert fluorescent microscope. Bar: 200 μm. (D): CCK-8 assay confirmed that MSC proliferation was not affected after lentivirus transduction. (E): Western blot confirmed that HIF-1α protein was highly-expressed in MSC^HIF-1α^ group. (F): HIF-1α mRNA expression level was significantly elevated after lentivirus transduction (*n* = 3).

**Additional file 2: Supplemental Fig. 2.** Characterization of mesenchymal stem cells–derived exosomes. (A): Nanoparticle tracking analysis (NTA) showed the particle size distribution of MSCs-derived exosomes. (B): Transmission electron microscope (TEM) images of MSC-derived exosomes. Bar: 100 nm. (C): Western blot analysis of MSC-derived exosomes by CD63 and TSG101. (D): HIF-1α mRNA expression level in exosomes derived from HIF-1α overexpressed MSCs measured by RT-qPCR (n = 3). ^***^P < 0.001.

## Data Availability

The data that support the findings of this study are available from the corresponding authors upon reasonable request.

## Abbreviations

MI: Myocardial infarction
MSCs: Mesenchymal stem cells
HIF-1α: Hypoxia-inducible factor 1-alpha
HUVECs: Human umbilical vein endothelial cells
BMMCs: Bone marrow-derived mononuclear stem cells
VEGF: Vascular endothelium growth factor
PDGF: Platelet-derived growth factor
Ang-1: Angiopoietin 1
NTA: Nanoparticle tracking analysis
TEM: Transmission electron microscope
PVDF: Polyvinylidene difluoride
PBS: Phosphate-buffered saline
H&E: Hematoxylin and eosin
HRE: Hypoxia response element
ROS: Reactive oxygen species
