# Supplementary figures and images for: HIF-1α overexpression in mesenchymal stem cell-derived exosomes mediates cardioprotection in myocardial infarction by enhanced angiogenesis
Source: Stem Cell Res Ther. 2020 Aug 28;11:373. doi: 10.1186/s13287-020-01881-7 (PMC7455909; doi:10.1186/s13287-020-01881-7)

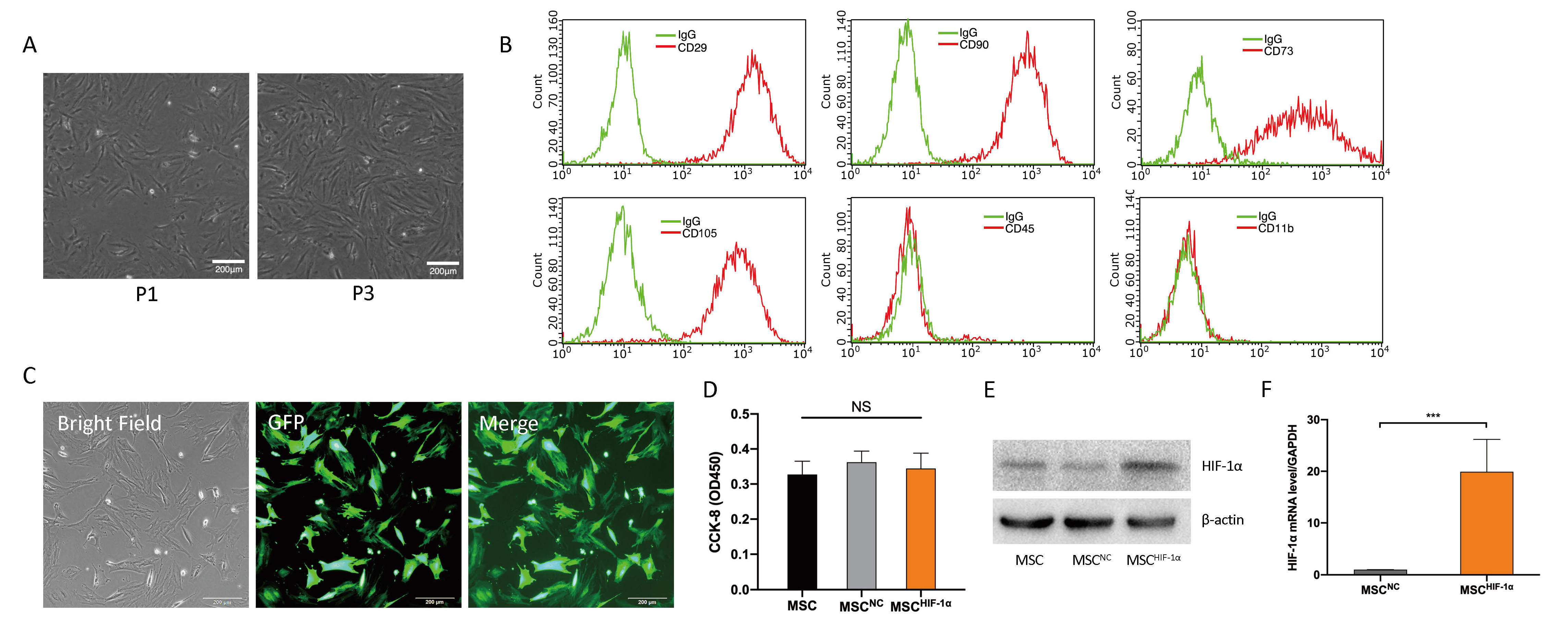

Supplement: Supplementary file 1 — Additional file 1: Supplemental Fig. 1. Characterization of mesenchymal stem cells (MSCs). (A): Morphology of MSCs (P1, P3) observed under microscope. Bar: 200 μm. (B): Cell surface antigens of MSCs, CD29, CD90, CD73, CD105, CD45 and CD11b, analyzed by flow cytometry. (C): Representative images of MSCs transduced with lentivirus containing HIF-1α were taken under an invert fluorescent microscope. Bar: 200 μm. (D): CCK-8 assay confirmed that MSC proliferation was not affected after lentivirus transduction. (E): Western blot confirmed that HIF-1α protein was highly-expressed in MSCHIF-1α group. (F): HIF-1α mRNA expression level was significantly elevated after lentivirus transduction (n = 3). [file 13287_2020_1881_MOESM1_ESM.jpg]

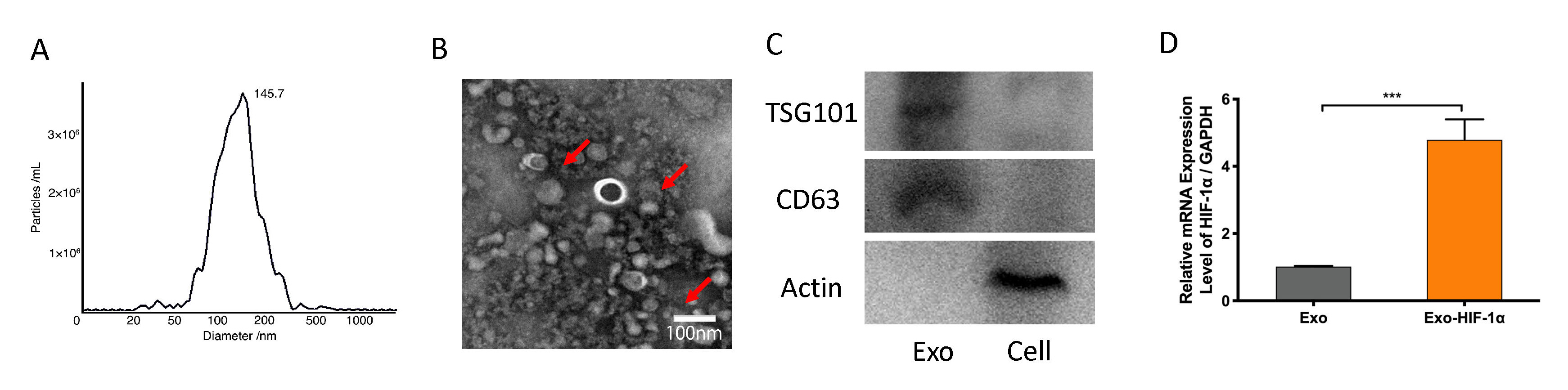

Supplement: Supplementary file 2 — Additional file 2: Supplemental Fig. 2. Characterization of mesenchymal stem cells–derived exosomes. (A): Nanoparticle tracking analysis (NTA) showed the particle size distribution of MSCs-derived exosomes. (B): Transmission electron microscope (TEM) images of MSC-derived exosomes. Bar: 100 nm. (C): Western blot analysis of MSC-derived exosomes by CD63 and TSG101. (D): HIF-1α mRNA expression level in exosomes derived from HIF-1α overexpressed MSCs measured by RT-qPCR (n = 3). ***P < 0.001. [file 13287_2020_1881_MOESM2_ESM.jpg]
